# Psychosocial and pharmacological interventions for the treatment of cannabis use disorder

**DOI:** 10.12688/f1000research.11191.1

**Published:** 2018-02-12

**Authors:** Pamela Sabioni, Bernard Le Foll

**Affiliations:** 1 Translational Addiction Research Laboratory, Campbell Family Mental Health Research Institute, Centre for Addiction and Mental Health , Toronto, Ontario , M5S 2S1, Canada; 2 Addiction Medicine Service, Centre for Addiction and Mental Health, Toronto, Ontario, M6J 1H4, Canada; 3 Department of Family and Community Medicine, Pharmacology and Toxicology, Psychiatry, Institute of Medical Sciences, University of Toronto, Toronto, Ontario, Canada; 4 Campbell Family Mental Health Research Institute, Centre for Addiction and Mental Health, Toronto, Ontario, Canada

**Keywords:** Cannabis, treatment, psychosocial, pharmacological, cannabinoids, CB1, CBD, human clinical trial

## Abstract

Cannabis use has been continuously increasing, and cannabis use disorder (CUD) has become a public health issue. Some psychosocial interventions have demonstrated the ability to reduce cannabis use; however, there are no pharmacotherapies approved for the treatment of CUD. Some drugs have shown limited positive effects on use and withdrawal symptoms, but no controlled studies have been able to show strong and persistent effects on clinically meaningful outcomes. The aim of this review is to synthesize the evidence from the available literature regarding the effectiveness of psychosocial and pharmacological treatments for CUD among adults (that is, 18 years old or older). An analysis of the evidence shows that the current best psychosocial intervention to reduce cannabis use is the combination of motivational enhancement therapy and cognitive-behavioral therapy, preferably accompanied by a contingency management approach. In regard to pharmacological interventions, there are mostly unclear findings. Some drugs, such as CB1 agonists, gabapentin, and N-acetylcysteine, have been shown to produce improvements in some symptoms of CUD in single studies, but these have not been replicated. Other classes of medications, including antidepressants and antipsychotics, have been unsuccessful in producing such effects. There is an imminent need for more clinical trials to develop more effective treatments for CUD.

## Introduction

Cannabis is one of the most commonly used drugs at the global level, and an estimated 183 million people used cannabis in 2014. Similar to other psychotropic drugs, cannabis has the potential to produce rewarding/reinforcing effects by enhancing dopamine signaling in the mesolimbic and mesocortical pathways ^1^. The rewarding effects of cannabis are directly associated with the actions of Δ9-tetrahydrocannabinol (THC) (the main psychoactive ingredient of cannabis) on cannabinoid CB1 receptors in the brain ^2, 3^.

Cannabis use has increased in North America over the past few years, most notably in jurisdictions that have legalized access. For example, in Colorado, cannabis use among young adults (18–25 years old) increased from 20% to 31% between 2000 and 2014 ^4^. It is estimated that about 8% to 9% of people who have used cannabis in their lifetime will develop cannabis dependence, corresponding to the most severe form of cannabis use disorder (CUD) ^5, 6^. An analysis of the numbers in treatment for cannabis use in the United States and Europe shows an increase over the long term ^7^. The increase in treatment demand is partially due to the availability of new high-potency strains of cannabis and synthetic cannabinoids ^8, 9^. THC levels have increased to as much as 20–25% over the past few years, whereas cannabis concentrate or synthetic cannabinoid products can contain up to 80–90% THC or more potent cannabinoid agonists ^10– 13^.

Cannabis use has been associated with several adverse effects ^9, 14^. Subjects can present for cannabis intoxication. The main symptoms are euphoria, but symptoms or signs can include increased appetite, tachycardia, tachypnea, and altered judgment, and there can be psychiatric complications (for example, anxiety and psychosis). Discontinuation of cannabis after regular, prolonged use is associated with a withdrawal syndrome that is recognized (anxiety, dysphoria, sleep disturbance, irritability, and anorexia).

Exposure to cannabis can induce medical complications, such as cardiovascular and respiratory problems, but also impact the function of the brain, reducing the ability to drive, decreasing cognitive function, and decreasing memory function ^9, 15– 17^. Additionally, early onset cannabis users have shown a clear increased risk of CUD development, and there may be alterations of white and gray brain matter and cortical thickness ^18– 21^. Frequent use of high-potency cannabis has been associated with increased paranoia, greater CUD severity, elevated risk for psychotic disorder, and cannabis-induced psychosis among individuals with no psychiatric history ^9, 22– 24^.

CUD is characterized in the fifth edition of the *Diagnostic and Statistical Manual of Mental Disorders* (DSM-5) by a pattern of cannabis use that causes clinically significant psychiatric distress and social impairment as well as multiple adverse consequences associated with cannabis use and repeated unsuccessful attempts to stop using ^25^. Cannabis use persists despite negative consequences, and most individuals with CUD perceive themselves as unable to quit ^26^. Epidemiological studies have estimated that around one in six of those who use cannabis during adolescence and one in two daily cannabis users will meet the criteria for CUD ^27, 28^.

Research has focused on interventions to decrease use, promote abstinence, and prevent relapse of cannabis use. Evidence from scientific and clinical literature shows that some psychosocial interventions might help decrease cannabis use, while pharmacotherapies have shown limited effectiveness to treat CUD. The aim of this narrative review is to summarize the evidence regarding the effectiveness of psychosocial and pharmacological treatment interventions for CUD, emphasizing recent advances for the past 3 to 4 years of research.

## Methods

The present narrative review on recent advances of psychosocial and pharmacological interventions for CUD followed a structured review approach, based on electronic searches for peer-reviewed publications in relevant scientific databases (that is, MEDLINE, Embase, PsycINFO, and the Cochrane Library). A search strategy was developed for MEDLINE and revised appropriately for all other databases. Keyword examples include “cannabis”, “marijuana”, “treatment”, “intervention”, “psychosocial”, “psychological”, “therapy”, “pharmacological”, “pharmacotherapy”, and “drug” as well as their related terms and variations. Studies presenting data specifically on psychosocial and pharmacological interventions for CUD among adults (18 years old or older) were considered for inclusion. Relevant data were extracted, synthesized, and summarized into a narrative approach.

## Discussion/analysis of the recent literature

### Psychosocial interventions for cannabis use disorder

There is strong support in the literature and clinical practice for psychosocial interventions for the management of CUD ^29, 30^. Most of the recent research on treatments for CUD involves either a combination of psychosocial and pharmacological interventions or pharmacological interventions alone. In the past, a number of clinical trials have explored the effectiveness of psychosocial interventions for CUD, and most studies focused on the effects of primary psychosocial interventions for CUD, such as cognitive-behavioral therapy (CBT) and motivation enhancement therapy (MET). Such interventions can be delivered individually or in groups and focus on the individual or the social environment, teaching coping strategies and problem-solving skills. In general, these psychosocial approaches for substance use disorders aim to build motivation, identify patterns of use and triggers that lead to use, and manage and promote substitution of substance-related behaviors with healthier activities ^31, 32^.

Studies have also investigated interventions with alternative approaches for the treatment of CUD. For example, mindfulness-based meditation is a technique that aims to enhance moment awareness in order to decrease the impact of triggers that lead to cannabis use ^33^. Additionally, drug counseling (DC) might be offered to promote education regarding drug use and health risks and provide suggestions to help decrease the harmful effects of drug use ^30^.

Most studies showed that CBT and MET present similar treatment effectiveness. Studies comparing either CBT or MET with alternative psychosocial interventions consistently found that these two therapies produce the greatest reduction in cannabis use. For example, a recent study showed that a combined CBT and MET approach reduced the frequency of cannabis use more effectively when compared with either intervention alone ^34^. One study reported that CBT produced superior effectiveness when compared with MET ^35^. Finally, MET demonstrated superior outcomes compared with drug-related health education (both at 6 and 12 months) ^36^ and with inactive controls ^37, 38^.

Studies show that the effects of the CBT + MET intervention are positively enhanced by contingency management (CM), a financial incentive for successful abstinence or treatment adherence. For example, CBT + MET enhanced by abstinence-based CM promoted superior results when compared with any other psychosocial approach, such as MET + CBT + adherence-based CM, CBT + adherence-based CM, abstinence-based CM alone, MET + CBT, and finally DC alone ^39– 41^.

However, one study showed that, when delivered alone, a 12-session CBT intervention showed greater reduction in the frequency of cannabis use compared with the same CBT duration paired with abstinence-based CM or adherence-based CM over 12 months ^42^.

In addition, longer or more intensive interventions have been shown to be superior to shorter durations of treatment. It has been demonstrated that a nine-session MET + CBT intervention outperformed a shorter two-session counterpart for up to 15 months ^36^. Additionally, six-session CBT produced superior results when compared with a single CBT session in another clinical trial ^25^.

In summary, the best intervention for reducing the frequency of cannabis use is likely to be a MET + CBT combination enhanced by abstinence-based CM when available. In the absence of CM, MET + CBT is likely to remain effective, although improvements may not be as immediately noticeable. Although the optimum number of sessions is not clear, evidence suggests that more intensive interventions of more than four sessions are likely to be superior to less intensive interventions, at least in the short term.

### Pharmacological interventions for cannabis use disorder

To date, there are no medications approved for the treatment of CUD. Human laboratory studies and clinical trials have tested multiple medications normally approved for other conditions, such as antidepressants, anxiolytics, mood stabilizers, and antiepileptic drugs ^43– 49^. For example, the anticonvulsant gabapentin, a GABA/calcium channel modulator, reduced cannabis use and withdrawal symptoms in adults with CUD ^47^. In a recent meta-analysis, selective serotonin reuptake inhibitor (SSRI) antidepressants, mixed-action antidepressants, atypical antidepressants (bupropion), anxiolytics (buspirone), and norepinephrine reuptake inhibitors (atomoxetine) did not demonstrate effects that would suggest that these medications would help for CUD treatment ^50^.

Recently, more attention has been given to medications that might act as substitution therapy for CUD, following the same principle as methadone for the treatment of opioid use disorder or nicotine replacement therapy for tobacco dependence. The aim of agonist replacement therapy is to reduce the desire to use the drug, withdrawal symptoms, and the positive subjective and reinforcing effects that are directly mediated by receptor activation.

In the past, THC formulations have been assessed for CUD and have shown some mixed outcomes in both randomized clinical trials and laboratory studies. For example, although the CB1 agonist dronabinol has reduced cannabis withdrawal symptoms and increased retention in treatment when compared with placebo in early studies ^51^, dronabinol produced no greater effects than placebo when combined with lofexidine (an agonist of the α2-adrenergic receptor) for cannabis abstinence in a recent clinical trial ^52^.

Cannabidiol (CBD) is the second major component of marijuana and has gained high visibility for its multiple therapeutic properties. The mechanism of action of CBD at the CB1 receptor is not yet clear, but recent discussion suggests that it decreases CB1 activity probably through a negative allosteric activity without producing the side effects of inverse agonists ^53, 54^. To date, CBD has demonstrated anti-inflammatory, anticonvulsant, antipsychotic, anxiolytic, and neuroprotective effects in pre-clinical studies; some of those effects have been confirmed in human studies. Unlike THC, CBD does not produce psychotropic effects and this is probably because of its low affinity for CB1 receptors ^55– 57^. Studies have assessed the effects of CBD for the treatment of CUD, both alone and in a formulation combined with THC (nabiximols, Sativex). Recent studies report that the THC/CBD mixture does not elicit as much of a psychoactive effect as cannabis and, despite not eliciting greater reduction in cannabis use when compared with placebo, was able to reduce withdrawal symptoms and improve retention in treatment in both a clinical trial ^58^ and a human laboratory study ^59^. However, CBD alone was unable to reduce self-administration in a human laboratory study and did not alter the subjective and physiological effects of smoked marijuana ^60^.

Because the rewarding effects of cannabis are directly associated with the actions of THC on the CB1 receptors, CB1 receptor antagonists/inverse agonists also have been evaluated in human laboratory studies ^61, 62^. However, psychiatric side effects associated with CB1 antagonists/inverse agonists, such as depression- and anxiety-like states, have been reported in several studies, leading patients to drop out of studies and discontinue treatment ^63– 65^. The recent report that a neutral CB1 antagonist (AM4113) may retain the therapeutic potential of inverse agonists ^66, 67^, possibly without the neuropsychiatric side effects ^67^, provides some development opportunities in this area.

Recently, a number of trials have evaluated N-acetylcysteine, a modulator of glutamatergic receptors, because of positive results for cannabis use cessation and subjective measures (craving) in past open-label and placebo-controlled trials among adolescents ^68, 69^. However, its effect was not replicated in a larger clinical trial among adults, in which N-acetylcysteine and placebo groups did not differ in cannabis abstinence ^70^. The potential utility of N-acetylcysteine should be further evaluated in adolescents in the future.

There is currently substantial evidence that the cannabinoid system interacts with other neurotransmitters and neuromodulators. Opioid-focused medications have been considered a potential target for the treatment of CUD, since endogenous opioids play an important role in modulating the addictive properties of cannabinoids. Evidence from pre-clinical studies demonstrates that antagonists of opioid receptors were able to reduce discriminative stimulus and reinforcing effects produced by the activation of CB1 receptors ^71, 72^. In addition, studies have shown that cannabinoids produce clear opioid-sparing effects ^73^.

Additionally, some studies have been conducted on the effects of naltrexone, an antagonist of the µ-opioid receptor, on cannabis use. It has been reported that the acute administration of naltrexone potentiated the positive subjective effects of cannabis but that repeated administration decreased such effects ^74, 75^. This controversial effect dependent on the duration of treatment can be observed in other neurotransmitter systems. It has been noted that acute compared with chronic antagonism of the dopamine receptor elicits opposite effects on cocaine reinforcement in both human and non-human subjects ^76^.

Studies have found that a genetic variation of fatty acid amine hydrolase (FAAH), the enzyme responsible for the degradation of endocannabinoids, is associated with CUD ^77, 78^. Among subjects who tried cannabis, those carrying a genetic variation in FAAH (C385A) were significantly less likely to develop CUD ^79^. The variation C385A in FAAH reduces both the enzyme’s expression and its activity ^80^, and FAAH expression is reduced in subjects with CUD ^81^. An ongoing clinical trial is further exploring the effects of PF-04457845 (a FAAH inhibitor) on cannabis withdrawal ^82^.

Currently, there are several clinical trials assessing psychosocial and pharmacological interventions to reduce cannabis use and withdrawal symptoms and prevent relapse. These include trials with CBD ^83^, nabilone ^84^, CI-581a (a glutamate modulator) in combination with MET and mindfulness-based relapse prevention ^85^, and a combination of numerous behavioral therapies (MET, CBT, CM, individualized assessment, and treatment) ^86^. The outcomes from these clinical trials will improve our understanding of treatments for CUD.

## Conclusions

CUD is an increasingly important public health issue, and clinical research has been investing in potential treatments for CUD. To date, several studies have investigated psychosocial interventions and concluded that a combination of CBT and MET represents the best approach to treat CUD and that abstinence-based CM (incentives) can enhance effectiveness ^30^.

Recently, several pharmacological interventions have been investigated; however, only a few have shown encouraging results. Specifically, cannabinoid medications have most consistently demonstrated the ability to decrease withdrawal symptoms. However, their utility to reduce cannabis use and prevent relapse still needs further investigation.

For other substance use disorders (for example, alcohol, tobacco, and opiates), treatment guidelines usually recommend a combination of psychosocial and pharmacological interventions ^87^. Since this approach has not yet been validated for CUD, the improvement of psychosocial treatments with and without pharmacological therapies for CUD should be further explored in future clinical research.
